# Novel Long Non-coding RNA Expression Profile of Peripheral Blood Mononuclear Cells Reveals Potential Biomarkers and Regulatory Mechanisms in Systemic Lupus Erythematosus

**DOI:** 10.3389/fcell.2021.639321

**Published:** 2021-06-02

**Authors:** Qi Cheng, Mo Chen, Xin Chen, Xiaochan Chen, Huawei Jiang, Huaxiang Wu, Yan Du

**Affiliations:** ^1^Department of Rheumatology, School of Medicine, The Second Affiliated Hospital, Zhejiang University, Hangzhou, China; ^2^Department of Clinic Medicine, School of Medicine, The Second Affiliated Hospital, Zhejiang University, Hangzhou, China; ^3^Department of Hematology, School of Medicine, The Second Affiliated Hospital, Zhejiang University, Hangzhou, China; ^4^Cancer Institute, Key Laboratory of Cancer Prevention and Intervention, China National Ministry of Education, Key Laboratory of Molecular Biology in Medical Sciences, Zhejiang, China

**Keywords:** systemic lupus erythematosus, long non-coding RNA, gene expression profile, transcriptome sequencing, biomarker, regulatory mechanism

## Abstract

**Objective:**

The multisystem involvement and high heterogeneity of systemic lupus erythematosus (SLE) lead to great challenges in its diagnosis and treatment. The purpose of this study was to find new lncRNAs in peripheral blood mononuclear cells of SLE patients by transcriptome sequencing and explore their potential as biomarkers and their correlation with clinical features.

**Materials and Methods:**

Transcriptome sequencing was used to screen differentially expressed lncRNAs (DELs) and mRNAs (DEMs). The expression of these selected lncRNAs and mRNAs in SLE patients and healthy controls was verified by qPCR. DAVID and WebGestalt were used to perform enrichment analysis. Cytoscape was used to construct a protein–protein network, a coexpression network, and a competitive endogenous RNA network to reveal the regulatory mechanisms of lncRNAs at the transcriptome level.

**Results:**

A total of 1737 DELs and 4078 DEMs were identified between SLE patients and healthy controls. Ten lncRNAs and eight genes were verified by qPCR in a larger sample set. The lncRNA *NONHSAT101022.2* was significantly downregulated in SLE patients and was also significantly related to the activity and severity of disease. The upregulated genes were enriched in defense and the immune response, while the downregulated genes were mainly enriched in SLE-related pathways. Topology network analysis revealed that the lncRNAs were involved in regulation at the transcriptome level, including acting directly on mRNA or indirectly affecting gene expression by acting on miRNA.

**Conclusion:**

In this work, we identified many mRNAs and novel lncRNAs by transcriptome sequencing. The functions and regulatory mechanisms of these lncRNAs were analyzed by bioinformatic methods. The novel lncRNA *NONHSAT101022.2* is significantly downregulated in SLE patients and is significantly related to the activity and severity of disease. Additionally, we propose that *NONHSAT101022.2* may enhance the signal transduction of β2-AR by cis regulating *LMBRD2*, inducing NK cells to produce high levels of IFN-γ and thereby exacerbating SLE.

## Introduction

Systemic lupus erythematosus is a complex and heterogeneous autoimmune disease, usually involving multiple systems of the whole body. The health-related quality of life in SLE is significantly impaired (Carter et al., 2016). Its prevalence rate is approximately 30–50 per 100,000 people (Dörner and Furie, 2019). A variety of factors can affect SLE, such as genetic, environmental, immunoregulatory, hormonal and epigenetic factors (Han, 2012). The outlook for SLE patients has improved significantly with advances in research, but this improvement is insufficient to meet patient needs because SLE still results in premature mortality (Jorge et al., 2018; Durcan et al., 2019). To obtain better diagnostic and therapeutic methods, an in-depth understanding of the pathogenesis of SLE is required.

Long non-coding RNAs are RNA molecules greater than 200 nucleotides that do not encode proteins. They have a variety of functions, including participating in the regulation of gene expression, regulating the function of proteins, and altering the structure of the genome (Engreitz et al., 2016). As research advances, the function of lncRNAs in the immune system is gradually being discovered, and lncRNAs have been shown to be key regulators of gene expression in the immune system (Chen et al., 2017). Several lncRNAs, such as *NEAT1*, *GAS5*, *TUG1*, and *Linc00513*, are dysregulated in SLE and are involved in the pathogenesis of SLE (Zhang et al., 2016; Xu et al., 2018; Xue et al., 2018; Liu et al., 2020). These results suggest that lncRNAs could be potential biomarkers for disease diagnosis and treatment. However, our current understanding of SLE-related lncRNAs is still limited.

Here, we used full transcriptome sequencing and microarray analysis to detect the expression of mRNAs and novel lncRNAs in peripheral blood mononuclear cells (PBMC) of SLE patients and healthy controls. After screening for the differentially expressed mRNAs (DEMs) and lncRNAs (DELs), Gene Ontology (GO), and Kyoto Encyclopedia of Genes and Genomes (KEGG) enrichment analyses were performed to find the enriched functions and pathways. Then, a protein–protein interaction (PPI) network, a lncRNA–mRNA coexpression network, and a competitive endogenous RNA (ceRNA) network were constructed and visualized by Cytoscape. These networks help us better understand the functions and regulatory mechanisms of lncRNAs. Ultimately, qPCR was used to verify the selected lncRNAs and mRNAs, and receiver operating characteristic (ROC) curve analysis was used to assess the diagnostic ability of the lncRNAs.

In this work, we describe novel lncRNAs that may play an important role in the pathogenesis of SLE and that were discovered through full transcriptome analysis of PBMCs in SLE patients. This study provides the potential for an in-depth understanding of the mechanisms of SLE development at the transcriptome level and the development of new diagnostic and therapeutic approaches.

## Materials and Methods

### Patients and Healthy Controls

We recruited 77 SLE patients and 24 healthy controls for this study. All the SLE patients were selected from the Department of Rheumatology, the Second Affiliated Hospital of Zhejiang University School of Medicine from July 2020 to October 2020. All SLE patients met the American College of Rheumatology 1997 criteria and the Systemic Lupus International Collaborating Clinics 2012 criteria for SLE (Hochberg, 1997; Petri et al., 2012). The first validation set for all the selected lncRNAs and mRNAs contained 44 of the 77 SLE samples and the 24 healthy controls. The other 33 SLE samples were used in the expanded validation set for lncRNA *NONHSAT101022.2*. The expression of *NONHSAT101022.2* in all 77 SLE samples was used to analyze the correlation with clinical features. The clinical characteristics and laboratory tests of the 77 SLE patients are listed in Table 1. Five SLE patients and five healthy controls were randomly selected for RNA sequencing.

**TABLE 1:** Clinical characteristics of 77 patients with SLE.

Variable	*N*	median (range)	Variable	*N*	Positive rate, *n* (%)
**General features**			**Clinical features**		
Age (years)	77	38.1 (15 to 66)	Facial erythema	77	23 (29.9%)
Sex (Female/male)	69/8	/	Oral ulcer	77	7 (9.1%)
Course (Month),	77	85.3 (0.5 to 480)	Arthralgia	77	26 (33.8%)
SLEDAI	77	10.62 (0 to 26)	Fever	77	15 (19.5%)
**Laboratory test**			Proteinuria	77	24 (31.2%)
ESR (mm/h)	76	38.04 (2 to140)	Raynaud’s phenomenon	77	10 (12.9%)
CRP (mg/L)	77	10.34 (0.1 to136)	Photoallergic	77	4 (5.2%)
IgG (g/L)	75	13.95 (3.1 to 36.7)	Dropsy of serous cavity	77	36 (46.7%)
IgM (g/L)	75	0.91 (0.17 to 3.03)	Alopecia	77	16 (20.7%)
IgA (g/L)	75	2.49 (0.78 to 6.58)	Lupus nephritis	77	37 (48.1%)
IgG4 (g/L)	60	0.48 (0.01 to 2)	Neuropsychiatric lupus	77	5 (6.5%)
C3 (g/L)	75	0.56 (0.19 to 1.32)	**Autoantibodies**		
C4 (mg/L)	75	88.58 (17 to 304)	ANA	70	66 (94.3%)
24-h proteinuria (mg/24 h)	70	959.84 (3 to 8316)	Anti-dsDNA	70	42 (60.0%)
WBC (/L)	76	6.82 (2.8 to 15.7)	Anti-SSA	70	47 (67.1%)
Hb (g/L)	76	103.61 (62 to 146)	Anti-Ro52	70	43 (61.4%)
PLT (/L)	76	182.83 (10 to 838)	Anti-SSB	70	10 (14.3%)
Lymphocyte percentage (%)	68	21.53 (3.5 to 74.6)	Anti-Smith	70	14 (20.0%)
Total T cell (%)	68	64.19 (5.13 to 92.3)	Anti-RNP	70	26 (37.1%)
CD4 + /CD8 + T cell	68	1.03 (0.21 to 8.46)	Anti-RPP	70	35 (50.0%)
NK cell (%)	62	7.87 (1.3 to 43.94)	Anticardiolipin	70	10 (14.3%)
Total B/Lymphocyte (%)	62	16.45 (1.4 to 67.13)	Anti-nucleosome	70	34 (48.6%)
Treg (%)	62	3.68 (0.45 to 18.61)	Anti-histone	70	27 (38.6%)
Treg/CD4 + T cell (%)	62	6.99 (0.1 to 20.9)			

*SLEDAI, systemic lupus erythematosus disease activity index; ESR, erythrocyte sedimentation rate; CRP, C-reactive protein; C3/C4, complement 3/4; WBC, white blood cell; Hb, hemoglobin; PLT, platelet; Treg, regulatory T cells; ANA, antinuclear antibodies; SSA, single-stranded DNA; anti-SSB, anti RNA-protein complex antibodies; RNP, ribonucleoprotein; RPP, Ribosomal P protein.*

### RNA Extraction and Purification, Library Construction, and Next Generation Sequencing

Total RNA was extracted using the miRNeasy Mini Kit (Cat# 217004, Qiagen) following the manufacturer’s instructions, and an Agilent Bioanalyzer 2100 (Agilent Technologies, Santa Clara, CA, United States) was used to determine the RIN score to inspect the RNA integrity. Qualifying total RNA was further purified with the RNAClean XP Kit (Cat A63987, Beckman Coulter, Inc., Kraemer Boulevard Brea, CA, United States) and RNase-Free Dnase Set (Cat#79254, QIAGEN, GmBH, Germany).

According to the experimental instructions, rRNA removal, fragmentation, first strand cDNA synthesis, second strand cDNA synthesis, terminal repair, addition of 3′ terminal A overhang, connection, and enrichment were performed on the purified total RNA. Then the construction of the sequencing library was completed. The library was tested with a Qubit 2.0 Fluorometer and Agilent 4200 to determine its size.

An Illumina HiSeq 2000/2500 was used to complete the cDNA sequencing. Raw sequencing reads were filtered to obtain clean reads. The spliced mapping algorithm of HISAT2 (version 2.0.4) was used for genome mapping of the clean reads. StringTie (version 1.3.0) was used to count the fragments of each gene, and the TMM (trimmed mean of *M*-values) was used to normalize. Lastly, Perl scripts were used to calculate the Fragments Per Kilobase of transcript per Million mapped reads (FPKM) for each mRNA and lncRNA. EdgeR was used for gene analysis of intersample differences, and the Benjamini–Hochberg false discovery rate (FDR) method was used for multiple hypothesis testing and correction after the *P*-value was obtained (Benjamini and Hochberg, 1995; Benjamini and Yekutieli, 2001). The *Q*-value is the adjusted *P*-value. A | fold change| > 2 and a *Q*-value < 0.05 were the screening criteria for DEM and DEL.

### Prediction and Reannotation of Novel LncRNAs

The results from StringTie (version 1.3.0) were stitched with GffCompare (version 1.3.0), and the results were compared with the reference annotation to obtain new transcripts that did not match known annotations and to extract transcripts for lncRNA prediction. The specific steps were as follows: Step 1: transcript length ≥ 200 bp and exon ≥ 2; Step 2: predicted ORF < 300 bp; Step 3: predict Pfam, coding potential calculator (CPC) and coding–non-coding index (CNCI), take the intersection of the predicted results, and select transcripts with a CPC score < 0, a CNCI score < 0, and a Pfam with insignificant comparison as potential lncRNAs; Step 4: compared with known lncRNA, remove identical sequences (i: a transfrag falling entirely within a reference intron; u: an unknown, intergenic transcript; x: exonic overlap with reference on the opposite strand). The Perl script was used to find the genes corresponding to the novel lncRNAs on the chromosome, where for intronic and antisense lncRNAs the genes obtained corresponded to the lncRNA location and the genes at both ends of the lncRNA, and for intergenic lncRNA the genes nearest to the two ends of the lncRNA were obtained.

### Gene Ontology and Kyoto Encyclopedia of Genes and Genomes Enrichment Analysis

Two online tools, DAVID v 6.8^1^ and WebGestalt^2^ were used for GO and KEGG enrichment analyses (Huang da et al., 2009; Wang et al., 2017), the results of which were intersected with the next analysis. A *Q*-value < 0.05 was considered to represent a significant enrichment of differentially expressed genes, where the *Q*-value is the adjusted *P*-value.

### Protein–Protein Interaction Construction, Module Extraction, and Analysis

String v 11.0^3^ was used to complete the overall construction of the interactions between the proteins coded by the DEMs, and Cytoscape software was used for optimization. The MCODE and cytoHubba plugins of Cytoscape were used for further analysis. MCODE was used for module extraction, and cytoHubba was used to identify hub genes.

### DEL–DEM Coexpression Network Analysis

The Pearson Correlation Coefficient (PCC) between the DEMs and DELs was calculated to construct the DEL–DEM coexpression network, with a filter criterion of PCC > 0.995 (Schober et al., 2018).

### Construction of Competitive Endogenous RNA Network

The online tool AnnoLnc2^4^ was used to predict target miRNAs of selected lncRNAs. RNA22, DIANA-micro T, miRWalk, and miRDB were used to predict target miRNAs of selected lncRNA target genes. The intersection of the predicted results was taken as the target miRNAs. Cytoscape was used to construct and visualize the ceRNA network according to the interactive relationship between the lncRNAs, miRNAs, and mRNAs.

### Prediction of DEL Target Genes and LncRNA–DE Target mRNA Coexpression Network Analysis

To analyze the functions and mechanisms of these DELs, we predicted their target genes by cis-regulation and trans-regulation analysis (Ulitsky and Bartel, 2013). The trans-action prediction was based on the species mRNA database. Blast was first used to select sequences with complementary or similar characteristics, and RNAplex was used to calculate the complementary energy between the two sequences to select sequences above the threshold (Tafer and Hofacker, 2008). Genes less than 10 kb from the lncRNA were selected as target genes for cis action. Cytoscape software was then used to construct a DEL–DE target mRNA coexpression network. This network was constructed through cis and trans regulated effects and positive and negative regulated actions. The Cytoscape plugin ClueGO + CluePedia was used for the KEGG pathway enrichment analysis.

### Real-Time Quantitative Reverse Transcription-Polymerase Chain Reaction

Peripheral blood of the subjects was collected and used to extract PBMC. The total RNA of the PBMCs was extracted by Trizol. Takara reagents were used for genomic removal of total RNA, reverse transcription, and fluorescence quantitative PCR. Quantitative PCR analysis was performed on a 7500 Fast Real-Time PCR system (Applied Biosystems) using a TB Green PCR protocol. The selected lncRNAs and mRNAs were verified by qPCR and GAPDH as an internal reference. Their primer sequences are shown in Table 2. The 2^–ΔΔ*Ct*^ method was used to analyze gene expression.

**TABLE 2:** Primer sequences of selected lncRNAs and mRNAs.

Transcripts		Sequence
GADPH	Forward	AAGGTGAAGGTCGGAGTCAA
	Reverse	AATGAAGGGGTCATTGATGG
ENST00000625135	Forward	AGGGTTGGACTCGAGATGGA
	Reverse	ACCCCATGGCTCTGCTAAAC
MSTRG.44469.23	Forward	AAAACCTGCCATCCCCTCAG
	Reverse	GGCAAGCACACAGATGTTGG
NONHSAT227219.1	Forward	CCTACACAGCCCAACCATGT
	Reverse	CTTAAGGAAGGGCTGCCGAA
MSTRG.101921.8	Forward	GCTTCCTTGGCTGACGAGTT
	Reverse	TGTAGCCGAGAGCAGACACT
NONHSAT229190.1	Forward	GGACACCAGGTGGATGACTC
	Reverse	ATGTGAGCTTGCTGTGTCCA
NONHSAT037217.2	Forward	AGAGATCGGGTAGCTGCGGAAC
	Reverse	CAAAACGCCTGTGACCCCAAAAG
NONHSAT027287.2	Forward	TGAGCCATTGCTGATAGACTGTGC
	Reverse	AGATCCACTGCATAGTCCCAGAGC
NONHSAT248662.1	Forward	GCATTTGCCAGCCAGTTTCTATGG
	Reverse	TGTGCTACTGTGGGTCTCTTCCTG
NONHSAT101516.2	Forward	GGGTCACTGGATGACTTCATGTGG
	Reverse	CACTCGGGAACAGCAGCAAGG
NONHSAT101022.2	Forward	TGACCTCAATGGACCAATGGCTTG
	Reverse	CTGCTCTGTCTTACTTCCCACTGC
TMEM106B	Forward	ACACAGTACCTACCGTTATAGCA
	Reverse	TGTTGTCACAGTAACTTGCATCA
LRRN3	Forward	ACCAATGCTGCTCCTGACCAATG
	Reverse	GGTTGCCACAGTACCCCTTGAAG
CEP290	Forward	GCTTGGTGGTTGCGGTAGTGAG
	Reverse	GGCAGGTCATCTGGGTCAACTTTC
KLRC4	Forward	GCACAGTCCCTGACATCACACAG
	Reverse	CTTTGCTGCCTCTTTGGGTCCTG
CEACAM8	Forward	CCACCACTGCTCAGCTCACTATTG
	Reverse	AGTTGTAGCCACGAGGGTCCTG
MPO	Forward	TGGTGGGAGAACGAGGGTGTG
	Reverse	CGGTGGTGATGCCTGTGTTGTC
OLFM4	Forward	TAGGCAGCGGAGGTTCTGTGTC
	Reverse	AATTCCAAGCGTTCCACTCTGTCC
CEACAM6	Forward	AGGTGGACAGAGAAGACAGCAGAG
	Reverse	TGGCAGTGGTGGGTGGGTTC

### Statistical Analysis

IBM SPSS Statistics 25 (IBM Corp., Armonk, NY, United States) was used to analyze the data and draw the ROC curve. The area under the ROC curve (AUC) was used to assess the diagnostic value of the DELs in SLE. GraphPad Prism 8.0 (GraphPad Software, Inc., San Diego, CA, United States) was used to draw scatter diagrams. Student’s *t*-test was used to compare the differences between the two groups. *P* < 0.05 was considered statistically significant.

## Results

### Summary of RNA Sequencing and Characteristics of LncRNAs and mRNAs

**FIGURE 1:**
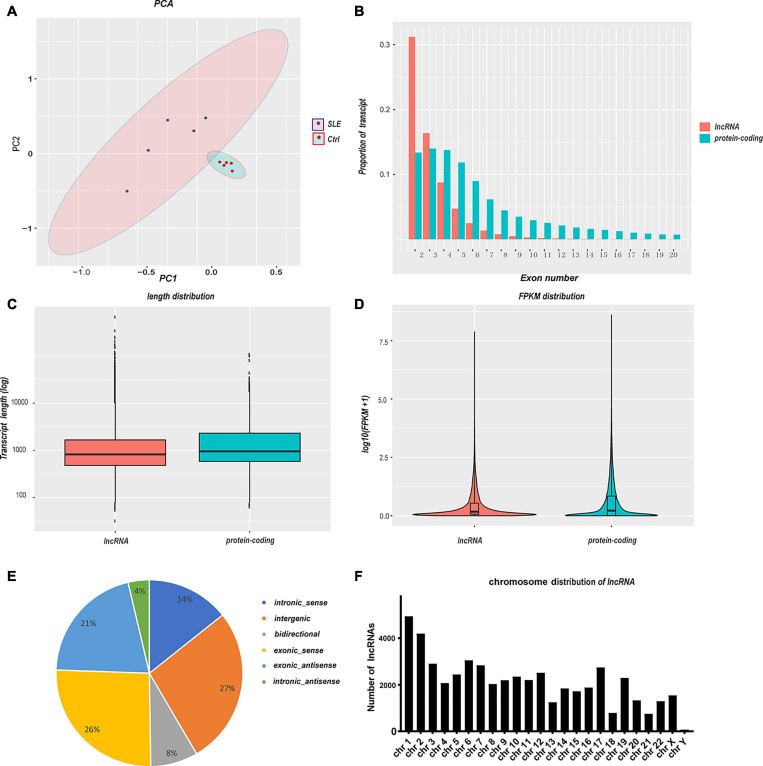
Summary of RNA sequencing and characteristics of lncRNAs and mRNAs. **(A)** Principal component analysis of SLE patients and healthy controls. The larger the variation between the two groups, the more significant the resulting data. **(B–D)** Comparison of lncRNAs and protein-coding genes in transcriptome sequencing. The lncRNAs showed a lower transcript proportion when **(B)** the exon number was greater than three, **(C)** the transcript length was shorter, and **(D)** the transcription abundance was inferior. **(E)** The classification of novel lncRNAs according to their genomic positional relationship with nearby mRNAs. **(F)** The chromosome distribution of novel lncRNAs.

Transcriptomic analysis was carried out to assess the differences in RNA expression between the SLE and control groups. The RNA-Seq reads were evaluated with the sequencing quality *Q*-value. The sequencing results for all samples were of high quality and the base distribution was balanced. The RNA-Seq produced an average of more than 70 million raw reads in SLE patients and the control group (Table 3). After filtering, we got over 94.49% clean reads for the two groups. Over 97.41% of mapping reads matched perfectly to the reference human genome. A total of 102,144 transcripts were detected in the RNA-Seq, of which 50,870 transcripts were identified as mRNA and 51,274 transcripts as novel lncRNA (after removing sequences of known lncRNA). The correlation coefficient and principal component analysis were used to check the relationship between samples (Figure 1A). The variation between the two groups was large, and the resulting data were significant. Moreover, the expression profile characteristics of lncRNAs and protein-coding genes were analyzed. Compared with the protein-coding genes, the lncRNAs showed a lower proportion of transcript when the exon number was greater than three (Figure 1B), a shorter transcript length (Figure 1C), and an inferior transcription abundance (Figure 1D). We can conclude that the predicted novel lncRNAs conform to the general characteristics of classical lncRNA. Next, we classified these novel lncRNAs according to their genomic positional relationship with nearby mRNAs. These novel lncRNAs were primarily intergenic (27%), exonic sense (26%), and exonic antisense (21%), and others included intronic sense (14%), bidirectional (8%), and intronic antisense (4%) (Figure 1E). The chromosome distribution of novel lncRNAs was discrepant (Figure 1F). Most of the novel lncRNAs were located on chromosome 1 and chromosome 2.

**TABLE 3:** Summary of draft reads by RNA sequencing.

Sample	Raw reads	Clean reads	Clean ratio	All genome mapping reads	Mapped reads	Mapping ratio
SLE 1	75011870	71689372	95.57%	70230692	68772515	97.92%
SLE 2	77308604	74619183	96.52%	73267266	71642345	97.78%
SLE 3	78367462	75368208	96.17%	73997158	72874853	98.48%
SLE 4	74556054	71473804	95.87%	70128268	68527225	97.72%
SLE 5	66562068	63641026	95.61%	62150876	60613397	97.53%
Ctrl 1	79039836	75467400	95.48%	73066362	71813055	98.28%
Ctrl 2	69375558	65550252	94.49%	63469462	62455181	98.40%
Ctrl 3	66445434	63955522	96.25%	62586114	61551228	98.35%
Ctrl 4	73887890	71089703	96.21%	69622918	68473265	98.35%
Ctrl 5	87846586	83862587	95.46%	80964202	78864444	97.41%

*Clean ratio = (Clean reads/Raw reads)%, Mapping ratio = (Mapped reads/All genome mapping reads)%.*

### Differentially Expressed mRNAs and LncRNAs in the SLE and Healthy Control Groups

**FIGURE 2:**
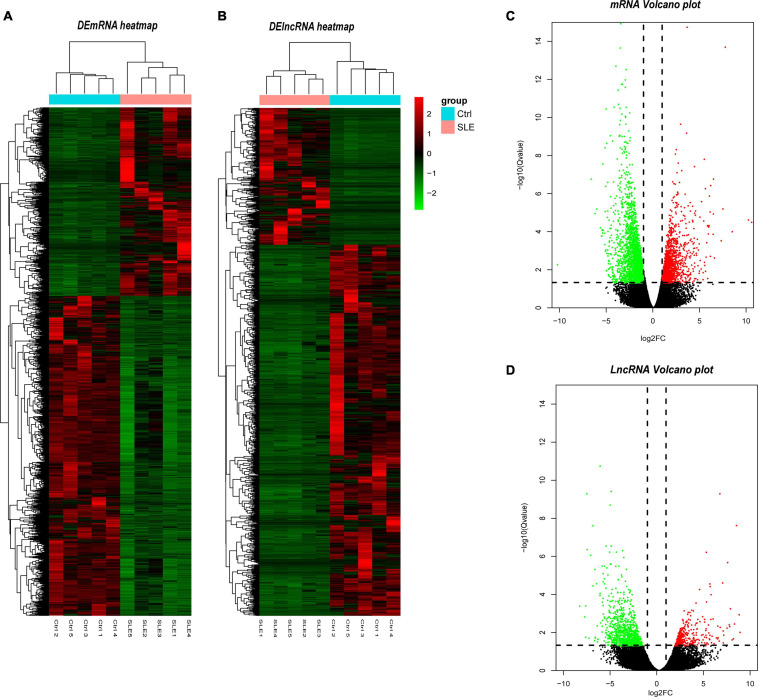
Identification of differentially expressed lncRNAs and mRNAs. **(A)** Heatmap of DELs between SLE and healthy control group. **(B)** Heatmap of DEMs between SLE and healthy control group. **(C)** Volcano plot of DELs between SLE and healthy control group. **(D)** Volcano plot of DEMs between SLE and healthy control group. In the hierarchical clustering heatmaps, green rectangles in each row represent low expression, and red rectangles represent high expression. The blue rectangle represents the control group and the pink rectangle represents the SLE group. In the volcano plot, the red dots represent upregulated DELs and the green dots represent downregulated DELs, the vertical dotted lines represent log_2_FC = 2 and -2, and the parallel dotted line represents *Q* < 0.05. The *Q*-value is the adjusted *P*-value.

The filter criteria for DEMs and DELs were as follows: (1) *Q*-value < 0.05, (2) fold change > 2. We identified 4078 DEMs and 1737 DELs. Among these DEMs, 1508 mRNAs were upregulated, and 2570 mRNAs were downregulated. The top five upregulated mRNAs were *TCN2*, *ADAMTS2*, *CTD-3222D19.4*, *EPHB2*, and *PLBD1*. The top five downregulated mRNAs were *NOG*, *SLC4A10*, *LRRN3*, *B3GALT2*, and *EEA1*. Furthermore, there were 468 upregulated lncRNAs and 1269 downregulated lncRNAs. The most upregulated lncRNAs were *MSTRG.75541.40*, *ENST00000620266*, *NONHSAT203045.1*, *MSTRG.56208.2*, and *MSTRG.97415.3*, and the most downregulated lncRNAs were *NONHSAT258765.1*, *NONHSAT137441.2*, *NONHSAT112119.2*, *NONHSAT209364.1*, and *NONHSAT227349.1*. To visualize these DEMs and DELs, hierarchical clustering heat map analysis (Figures 2A,B) and volcano plot analysis (Figures 2C,D) were performed.

### Verification of the Selected LncRNAs and mRNAs and ROC Curve Analysis of DELs

**FIGURE 3:**
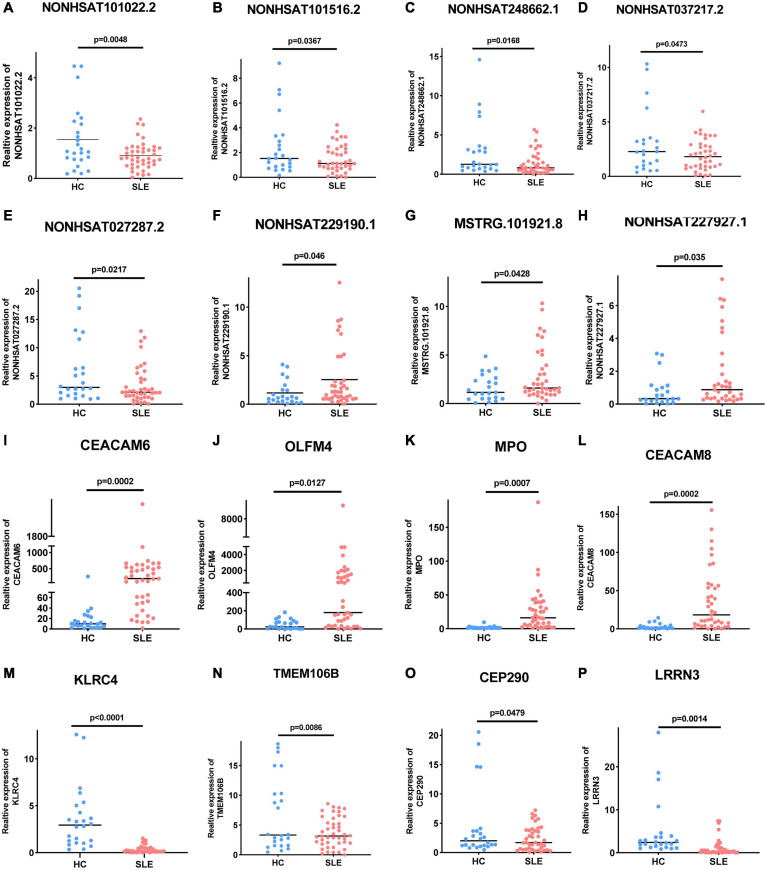
Expression of these selected lncRNAs and mRNAs in 44 SLE patients and 24 healthy controls. **(A–E)** Five selected lncRNAs, **(A)** *NONHSAT101022.2* (*P* = 0.0048), **(B)** *NONHSAT101516.2* (*P* = 0.0367), **(C)** *NONHSAT248662.1* (*P* = 0.0168), **(D)** *NONHSAT037217.2* (*P* = 0.0473), and **(E)** *NONHSAT027287.2* (*P* = 0.0217) were significantly downregulated in SLE patients. **(F–H)** Three selected lncRNAs, **(F)** *NONHSAT229190.1* (*P* = 0.046), **(G)** *NONHSAT037217.2* (*P* = 0.0428), and **(H)** *MSTRG.101921.8* (*P* = 0.035) were significantly upregulated in SLE patients. **(I–P)** Differential expression of mRNAs in SLE patients. **(I)** *CEACAM6* (*P* = 0.0002), **(J)** *OLFM4* (*P* = 0.0127), **(K)** *MPO* (*P* = 0.0007), and **(L)** *CEACAM8* (*P* = 0.0002) were significantly upregulated in SLE patients, while **(M)** *KLRC4* (*P* < 0.0001), **(N)** *TMEM106B* (*P* = 0.0086), **(O)** *CEP290* (*P* = 0.0479), and **(P)** *LRRN3* (*P* = 0.0014) were significantly downregulated. *P* < 0.05 was considered significant.

**FIGURE 4:**
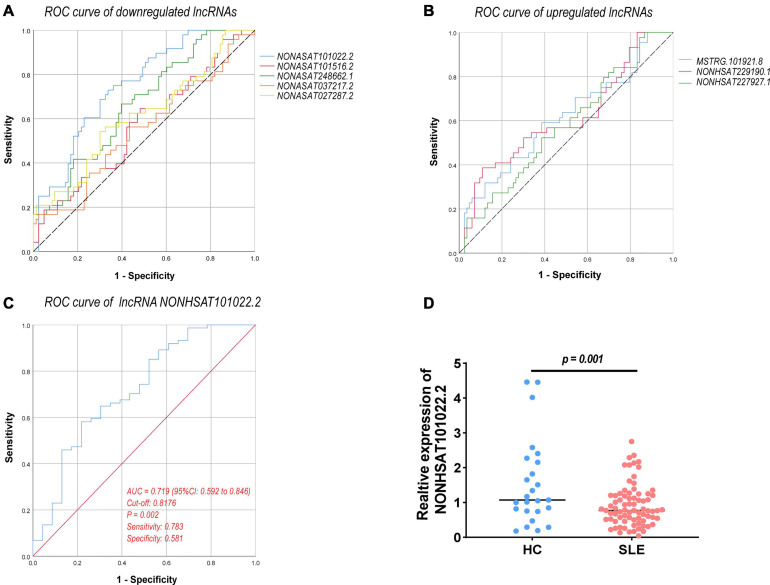
ROC curve analysis of verified DELs. **(A)** ROC curve of downregulated lncRNAs. **(B)** ROC curve of upregulated lncRNAs. **(C)** ROC curve of lncRNA *NONHSAT101022.2* (77 SLE patients vs. 24 healthy controls, AUC = 0.719, 95% CI: 0.592–0.846, *P* = 0.002, sensitivity: 0.783, specificity: 0.581). **(D)** Expression of *NONHSAT101022.2* in 77 SLE patients and 24 healthy controls (*P* = 0.001). ROC, receiver operating characteristic; AUC, the area under the ROC curve; CI, confidence intervals. *P* < 0.05 was considered significant.

Long Non-coding RNAs and mRNAs were selected for validation according to the following criteria: (1) No gene expression value was 0 in each sequenced sample; (2) The gene expression does not differ greatly between different samples, and the homogeneity is good. (3) There were significant differences between the two groups (according to the *Q*-value and fold change). Finally, top ten lncRNAs (five upregulated and five downregulated) and top eight mRNAs (four upregulated and four downregulated) were selected for further verification. We firstly expanded the samples to 44 SLE patients and 24 healthy controls. Among them, except for two upregulated lncRNAs, the rest were in line with the sequencing results. A scatter diagram is shown in Figures 3A–H and Supplementary Figure 1. Compared with healthy controls, the lncRNAs *NONHSAT037217.2*, *NONHSAT027287.2*, *NONHSAT248662.1*, *NONHSAT101516.2*, and *NONHSAT101022.2* were significantly downregulated in SLE patients, whereas the lncRNAs *NONHSAT229190.1*, *NONHSAT037217.2*, and *MSTRG.101921.8* were significantly upregulated. Unfortunately, we found no significance for the lncRNAs *ENST00000625135* and *MSTRG.44469.23* between the two groups. Consistent with the sequencing results, *CEACAM6*, *MPO*, *OLFM4*, and *CEACAM8* were significantly upregulated, and *KLRC4*, *CEP290*, *LRRN3*, and *TMEM106B* were significantly downregulated in SLE patients (Figures 3I–P). These results also indicate that the full transcriptome sequencing results are credible. To understand the diagnostic value of these eight lncRNAs in SLE, we drew the ROC curves (Figures 4A,B) and calculated the AUC values (Table 4). The lncRNA *NONHSAT101022.2* had a good diagnostic value for SLE (AUC: 0.747, 95% CI: 0.664–0.830, *P* = 0.000), while others had only general diagnostic value (an AUC between 0.5–0.7). The lncRNAs with an AUC greater than 0.6 were *NONHSAT248662.1* (AUC: 0.664, 95% CI: 0.570–0.757, *P* = 0.002), *NONHSAT027287.2* (AUC: 0.613, 95% CI: 0.511–0.716, *P* = 0.031), *NONHSAT229190.1* (AUC: 0.611, 95% CI: 0.504–0.718, *P* = 0.04), and *MSTRG.101921.8* (AUC: 0.609, 95% CI: 0.503–0.715, *P* = 0.044). Therefore, we selected *NONHSAT101022.2* for further analysis.

**TABLE 4:** ROC curve analysis of differentially expressed lncRNAs.

LncRNA	AUC	95%CI		Sensitivity	Specificity	Significance (*p*)	Cut-off
		
		Lower	Upper				
NONHSAT101022.2	0.747	0.664	0.830	9	0.662	*0.000*	1.159
NONHSAT101516.2	0.571	0.469	0.674	0.646	0.512	*0.176*	1.195
NONHSAT248662.1	0.664	0.57	0.757	0.667	0.602	*0.002*	1.067
NONHSAT037217.2	0.549	0.445	0.654	0.167	0.975	*0.349*	6.081
NONHSAT027287.2	0.613	0.511	0.716	0.542	0.699	*0.031*	3.055
MSTRG.101921.8	0.609	0.503	0.715	0.591	0.615	*0.044*	0.757
NONHSAT229190.1	0.611	0.504	0.718	0.386	0.892	*0.040*	0.810
NONHSAT227927.1	0.574	0.470	0.678	0.523	0.615	*0.170*	0.357

*ROC curve, receiver operating characteristic curve; AUC, area under the ROC curve; CI, confidence interval.*

### The Relationship Between *NONHSAT101022.2* and the Clinical Characteristics and Laboratory Tests of All 77 SLE Patients

**FIGURE 5:**
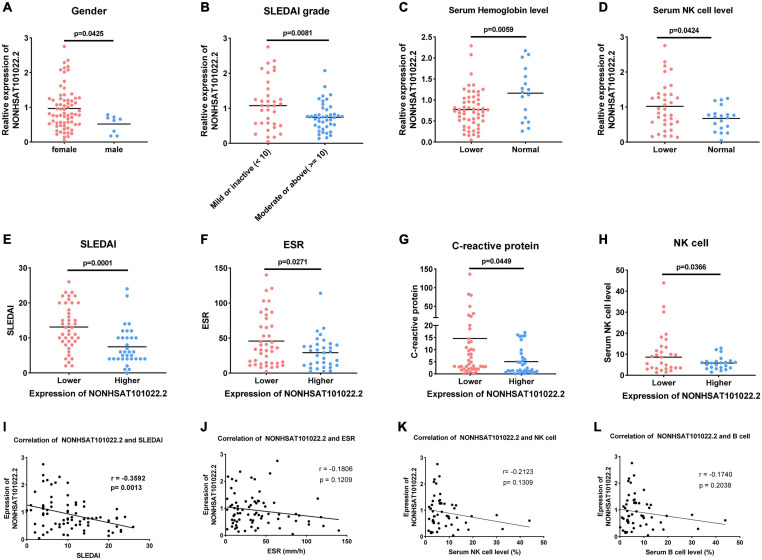
Relationship between lncRNA *NONHSAT101022.2* and clinical characteristics and laboratory tests of 77 SLE patients. **(A)** Compared with female patients, the expression of *NONHSAT101022.2* was significantly decreased in male patients. **(B–D)** Grouped according to clinical indicators, the expression of *NONHSAT101022.2* was significantly decreased in panel **(B)** the higher SLEDAI group (SLEDAI ≥ 10, *P* = 0.0001), **(C)** the lower hemoglobin group (Hb < 113 g/L (female) or Hb < 131g/L (male), *P* = 0.0058), and **(D)** the higher NK cell level group (NK cells > 7%, *P* = 0.0424). **(E–H)** Patients were divided into two groups according to the cutoff value (0.8176) at the optimal specificity and sensitivity in the ROC curve. The lower the expression of *NONHSAT101022.2*, the higher **(E)** the SLEDAI (*P* = 0.0081), **(F)** the ESR (*P* = 0.0271), **(G)** the CRP (*P* = 0.0449), and **(H)** the NK cell levels (*P* = 0.0366). **(I)** *NONHSAT101022.2* was significantly negatively correlated with the SLEDAI (*r* = –0.3592, *P* = 0.0013). **(J)** *NONHSAT101022.2* was negatively correlated with the ESR (*r* = –0.1806), but there was no significant difference (*P* = 0.1209). **(K)** *NONHSAT101022.2* was negatively correlated with the NK cell level (*r* = –0.2123), but there was no significant difference (*P* = 0.1309). **(L)** *NONHSAT101022.2* was negatively correlated with the ESR (*r* = –0.174), but there was no significant difference (*P* = 0.2308). *P* < 0.05 was considered significant.

The lncRNA *NONHSAT101022.2* was selected for further analysis. First, we examined the expression of *NONHSAT101022.2* in another 33 samples. The ROC curve and the scatter diagram of the lncRNA *NONHSAT101022.2* are shown in Figures 4C,D. Next, we analyzed the relationship between *NONHSAT101022.2* and the clinical characteristics and laboratory tests of all 77 SLE patients. Compared with female patients, the expression of *NONHSAT101022.2* was significantly lower in male patients (Figure 5A). When grouped according to clinical indicators, the expression of *NONHSAT101022.2* was significantly decreased in the group with a higher Systemic Lupus Erythematosus Disease Activity Index (SLEDAI) (Figure 5B), the group with lower hemoglobin (Figure 5C), and the group with higher NK cell levels (Figure 5D). Additionally, we divided patients into two groups according to the cutoff value (0.8176) at the optimal specificity and sensitivity in the ROC curve (Table 5). We found that the expression of the lncRNA is closely related to disease activity and severity. As the expression of *NONHSAT101022.2* decreased, the SLEDAI, Erythrocyte Sedimentation Rate (ESR), C reactive protein (CRP), and NK cell levels (Figures 5E–H) increased. Finally, we investigated the correlation between the expression of the lncRNA and several clinical indicators. The expression of *NONHSAT101022.2* showed a significant negative correlation with the SLEDAI (Figure 5I, *r* = –0.3592, *P* = 0.0013). The expression of *NONHSAT101022.2* was negatively correlated with ESR (Figure 5J, *r* = *–*0.1806, *P* = 0.1209), NK cell level (Figure 5K, *r* = –0.2123, *P* = 0.1309), and B cell level (Figure 5L, *r* = –0.174, *P* = 0.2308), but unfortunately there were no statistically significant differences. The reason for this may be that our sample size was relatively small. Consequently, prospective cohort studies with a larger sample size are needed to validate our findings in future.

**TABLE 5:** Relationship of the expression of the lncRNA *NONHSAT101022.2* with clinical features in SLE patients.

Variable	Higher expression, SLE (34)	Lower expression, SLE (43)	*P-*value
			
	Median (range)	Median (range)	
**General features**			
Age (years)	34.2 (15 to 61)	41.1 (15 to 66)	0.0294*
Sex (Female/male)	34/0	35/8	NS
Course (Month),	92.1 (1 to 480)	81.8 (0.5 to 480)	NS
SLEDAI	7.47 (0 to 24)	13.12 (2 to 26)	0.0001***
**Laboratory tests**			
ESR (mm/h)	29.18 (2 to 114)	45.67 (2 to140)	0.0271*
CRP (mg/L)	5.07 (0.1 to 17.1)	14.93 (0.1 to 136.3)	0.0449*
IgG (g/L)	13.85 (3.26 to 36.1)	14.28 (3.1 to 26.1)	NS
IgM (g/L)	0.88 (0.17 to 2.14)	0.94 (0.18 to 3.03)	NS
IgA (g/L)	2.34 (0.78 to 6.58)	2.62 (0.9 to 5.07)	NS
IgG4 (g/L)	0.41 (0.01 to 1.72)	0.51 (0.05 to 2)	NS
C3 (g/L)	0.59 (0.25 to 1.32)	0.54 (0.19 to 1.32)	NS
C4 (mg/L)	91.79 (17 to 352)	97.46 (18 to 304)	NS
24-h proteinuria (mg/24 h)	812.14 (7 to 4230)	1200.67 (0.22 to 8316)	NS
WBC (/L)	7.39 (2.4 to 18.5)	6.30 (1.7 to 16.5)	NS
Hb (g/L)	107.33 (68 to 146)	101 (62 to 134)	NS
PLT (/L)	193.58 (35 to 376)	176.07 (10 to 838)	NS
Lymphocyte percentage (%)	20.87 (6.23 to 35.2)	19.12 (3.48 to 74.58)	NS
Total T cell (%)	70.47 (50.6 to 92.3)	62.83 (5.43 to 90.6)	NS
CD4 + /CD8 + T cell	0.94 (0.45 to 1.72)	1.14 (0.21 to 8.46)	NS
NK cell (%)	5.95 (1.5 to 12.9)	10.16 (1.3 to 43.94)	0.0366*
Total B/Lymphocyte (%)	12.36 (2.6 to 32.6)	17.41 (1.4 to 67.13)	NS
Treg (%)	3.37 (0.51 to 9.8)	3.72 (0.45 to 18.61)	NS
Treg/CD4 + T cell (%)	6.44 (1.51 to 20)	7.8 (0.1 to 20.9)	NS

SLEDAI, systemic lupus erythematosus disease activity index; ESR, erythrocyte sedimentation rate; CRP, C-reactive protein; C3/C4, complement 3/4; WBC, white blood cell; Hb, hemoglobin; PLT, platelet; Treg, regulatory T cells. *P < 0.05 and ***P < 0.001.

### GO and KEGG Enrichment Analysis of Differentially Expressed Genes

**FIGURE 6:**
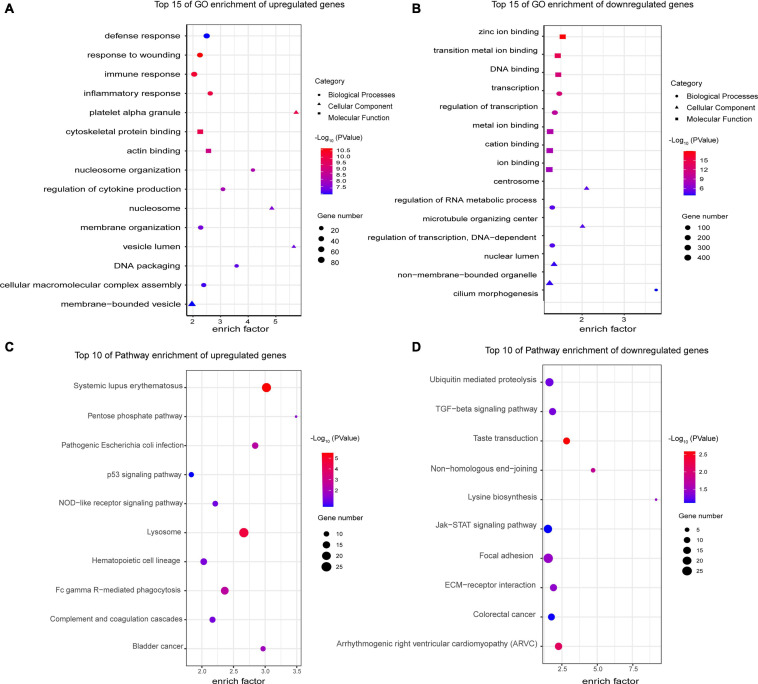
Gene Ontology and KEGG enrichment analyses of differentially expressed mRNAs. **(A)** Top 15 GO enrichment terms of upregulated genes. The most enriched terms were related to defense, immune and inflammatory response, which are the major characteristics of SLE. **(B)** Top 15 GO enrichment terms of downregulated genes. Their functions were mainly related to transcription and DNA binding. **(C)** Top 10 KEGG enrichment terms of upregulated genes. Most of the pathways play a critical role in the pathogenesis of SLE, such as Fc gamma R-mediated phagocytosis and the complement and coagulation cascades. **(D)** Top 10 KEGG enrichment terms of downregulated genes. The downregulated genes are involved in many significant pathways, such as the TGF-β and JAK-STAT signaling pathways. All GO and KEGG enrichment terms are screened based on a *Q*-value < 0.05.

Next, we analyzed the sequencing results as a whole using a variety of bioinformatics methods. We performed GO and KEGG enrichment analysis of the differentially expressed genes to analyze the function of these genes. The top 15 GO terms related to the upregulated and downregulated genes are shown in Figures 6A,B, respectively. Among the GO terms related to the upregulated genes, the most enriched terms were defense response (GO:0006952), response to wounding (GO:0009611), immune response (GO:0006955), inflammatory response (GO:0006954), and platelet alpha granule (GO:0031091). Most of them were related to immunity and inflammation. The most enriched terms for the downregulated genes were zinc ion binding (GO:0008270), transition metal ion binding (GO:0046914), DNA binding (GO:0003677), transcription (GO:0006350), and regulation of transcription (GO:0045449). For the KEGG enrichment analysis, we selected the top 10 pathway terms associated with the upregulated and downregulated genes to represent in bubble diagrams (Figures 6C,D). Most of the pathway terms for the upregulated genes were related to immune and inflammatory regulation of SLE, such as systemic lupus erythematosus (hsa05322), Fc gamma R-mediated phagocytosis (hsa04666), complement and coagulation cascades (hsa04610), and the NOD-like receptor signaling pathway (hsa04621). The downregulated genes were involved in many significant pathways, such as the transforming growth factor-β (TGF-β) signaling pathway (hsa04350) and the Janus kinase/signal transducer and activator of transcription (JAK-STAT) signaling pathway (hsa04630).

### Protein–Protein Interaction and DEL–DEM Coexpression Network Analysis

**FIGURE 7:**
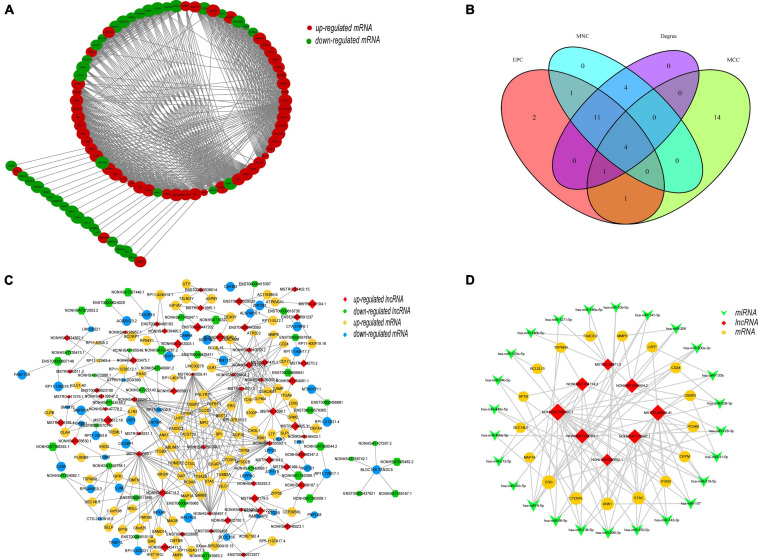
Topology network analysis of DEMs and DELs. **(A)** Protein–protein interaction subnetwork of DEMs. The gene cluster module with the highest score (score: 22.742), which has 90 nodes and 1012 edges, was extracted by the MCODE plugin. Each node represents a protein, while each edge represents one interaction between proteins. Red ellipse, upregulated mRNA; green ellipse, downregulated mRNA. **(B)** Identification of hub genes by four algorithms of the cytoHubba plugin, the MNC (Maximum Neighborhood Component), MCC (Maximal Clique Centrality), EPC (Edge Percolated Component), and Degree. Four hub genes, *CEACAM8*, *ELANE*, *ITGAM*, and *ITGB2*, were identified. **(C)** Coexpression network of DEMs and DELs. The coexpression network was constructed including 94 lncRNAs, 145 mRNAs, and 371 edges based on a PCC > 0.995 between DEMs and DELs. Each edge represents one interaction between a lncRNA and an mRNA. Red diamond, upregulated lncRNA; green hexagon, downregulated lncRNA; yellow ellipse, upregulated mRNA; blue ellipse, downregulated mRNA. **(D)** ceRNA network of selected lncRNAs, miRNAs and mRNAs. The ceRNA network consisted of 8 lncRNAs, 17 mRNAs and 25 miRNAs, reflecting the regulatory mechanism of the lncRNAs at the transcriptome level. Red diamond, lncRNA; green V, miRNA; yellow ellipse, mRNA.

Cytoscape was used to construct and visualize the PPI network, while the MCODE plugin was used to extract gene cluster modules. We selected the module with the highest score (score: 22.742), which had 90 proteins and 1012 edges and is shown in Figure 7A. Furthermore, we used another plugin of Cytoscape, cytoHubba, to identify the hub genes. Four of its algorithms—the Maximum Neighborhood Component, Maximal Clique Centrality, Edge Percolated Component, and Degree—were used to calculate the top 20 hub genes (Chin et al., 2014; Luan et al., 2020). The results of the four algorithms intersected and are shown in a Venn diagram (Figure 7B). We identified four hub genes: *CEACAM8*, *ELANE*, *ITGAM*, and *ITGB2*. Moreover, a coexpression network of DELs and DEMs was constructed, which comprised 94 lncRNAs, 145 mRNAs, and 371 edges (PCC > 0.995) (Figure 7C). Within the coexpression network, *MSTRG.4380.1* had the most coexpressed mRNAs (Park et al., 2009), and *DAP*, *HOMER2*, *PCSK6*, and *STAC* had the largest number of coexpressed lncRNAs (Chen et al., 2017). After incorporation of the predicted target miRNAs, we finally obtained 25 target miRNAs. A ceRNA network was constructed by Cytoscape, which consisted of 8 lncRNAs, 25 miRNAs, and 17 mRNAs (Figure 7D). These results reveal the regulatory mechanism of these lncRNAs at the transcriptome level. They can act directly on genes and regulate gene expression after interacting with miRNA.

### Analysis of Target mRNAs of DELs

**FIGURE 8:**
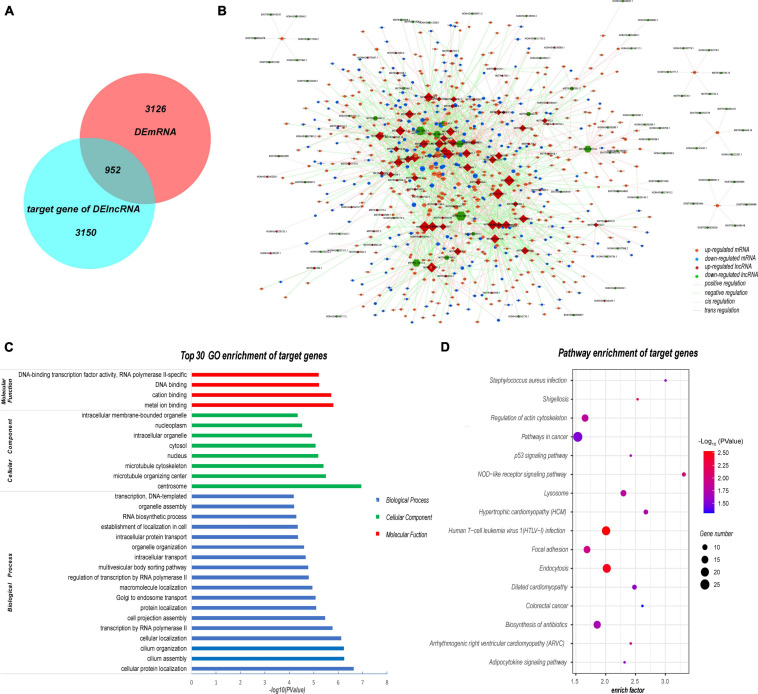
Analysis of target mRNAs of DELs. **(A)** Venn diagram showing that 952 of the 4102 target genes were differentially expressed in SLE patients. **(B)** Coexpression network of DELs and their target genes. The coexpression network consisted of 1575 nodes and 2269 edges. Each node represents a DEL or a mRNA, while each edge represents one interaction between DELs and DETGs. Red diamond, upregulated lncRNA; green hexagon, downregulated lncRNA; orange ellipse, upregulated mRNA; blue ellipse, downregulated mRNA. **(C)** Top 30 GO enrichment terms of target genes. Most of the DETGs were related to the nucleus and transcription regulation. **(D)** Pathway enrichment of DETGs. These pathway terms involved several pathways such as the NOD and p53 signaling pathways. All GO and KEGG terms were screened based on *Q* < 0.05.

**FIGURE 9:**
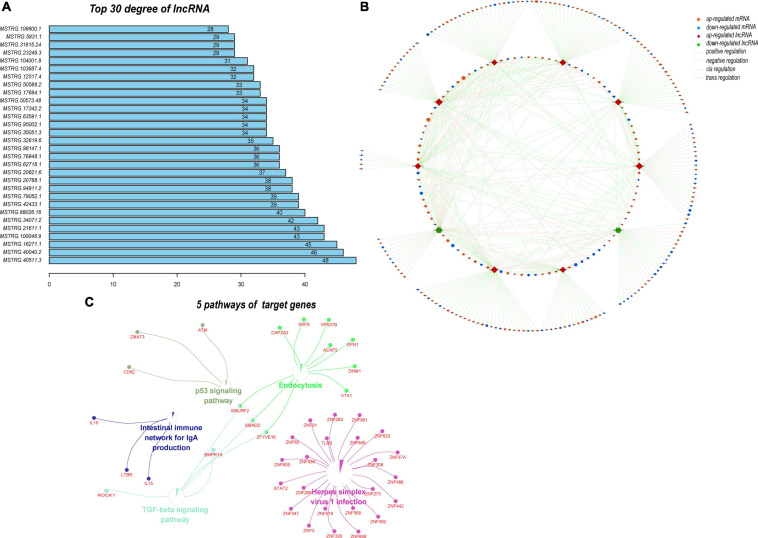
Analysis of the subnetwork of the 10 hub DELs. **(A)** Top 30 lncRNAs with the highest degrees in the DEL–DETG coexpression network. More important transcripts have a higher degree in the topology network. **(B)** Subnetwork of 10 hub DELs and their target genes. This subnetwork consisted of 10 lncRNAs, 279 mRNAs, and 423 edges. Each edge represents one interaction between DELs and DETGs. Red diamond, upregulated lncRNA; green hexagon, downregulated lncRNA; orange ellipse, upregulated mRNA; blue ellipse, downregulated mRNA. Pink line, positive regulation; green line, negative regulation; dashed line, cis regulation; solid line, trans regulation. **(C)** Five pathways of 279 DETGs. Subnetwork analysis helps us to better understand the function and mechanism of the important lncRNAs in the main network.

Next, we predicted the target genes of these DELs. A total of 4102 target genes were predicted and 10,569 lncRNA–target mRNA pairs were formed. By comparing the predicted target genes with the DEMs, 952 differentially expressed target genes (DETGs) and 2269 lncRNA–target mRNA pairs were obtained, and the results are shown by a Venn diagram (Figure 8A). On the basis of the interaction and regulation mode between the lncRNAs and target mRNAs, Cytoscape was used to construct a coexpression network (Figure 8B). Furthermore, GO and KEGG enrichment analyses were used to evaluate the function and pathway enrichment of 952 DETGs. The top 30 GO terms are shown in bar charts (Figure 8C); most of them related to transcription regulation, cilium development, and nucleus. The enrichment results of the pathway are shown by a bubble diagram (Figure 8D), including the NOD-like receptor signaling pathway, the p53 signaling pathway, and the adipocytokine signaling pathway. Through analysis of the network topology structure, the top 30 most connected lncRNAs were identified and are shown in the bar plot (Figure 9A). Subnetwork analysis enables us to better understand the function and mechanism of important lncRNAs in the main network. Therefore, we selected the top 10 lncRNAs as the hub lncRNAs to construct a hub lncRNA–DETG subnetwork (Figure 9B). The subnetwork consisted of 10 lncRNAs, 279 mRNAs, and 423 edges. Next, ClueGO + CluePedia, a plugin of Cytoscape, was used for the KEGG pathway enrichment analysis of mRNAs in the subnetwork. Five pathways were identified as enriched in the target genes (Figure 9C), including Herpes simplex virus type 1 infection, the TGF-beta signaling pathway, the intestinal immune network for IgA production, endocytosis, and the p53 signaling pathway.

## Discussion

The involvement of multiple organs in multiple systems of the whole body is an important characteristic of SLE, which leads to the complexity and heterogeneity of the pathogenesis of SLE. Genetic factors contribute to the development of SLE (Han, 2012; Rullo and Tsao, 2013), and the regulation of gene expression is key. The functional diversity of lncRNAs gives them the ability to participate in various aspects of the immune response (Yao et al., 2019). However, only a small percentage of lncRNAs have been recognized, and of those the function of only a small percentage have been found at present. Identifying and distinguishing novel and functional lncRNAs are important directions for future studies.

Here, a total of 1737 DELs including 468 upregulated and 1269 downregulated were identified in PBMCs of SLE patients by full transcriptome sequencing. The verification results of 8 of the 10 lncRNAs and all the mRNAs were consistent with the sequencing results. This shows that our full transcriptome sequencing is very reliable.

Because *NONHSAT101022.2* has a good diagnostic value for SLE, the function of lncRNAs may be determined by their target genes (Chen et al., 2017). Therefore, we carefully investigated *LMBRD2* (LMBR1 domain containing 2), a target gene of *NONHSAT101022.2*, which was also significantly downregulated in SLE patients in the RNA sequencing (healthy controls vs. SLE patients: 9.029 ± 1.274 vs. 1.177 ± 0.882, *P* < 0.0001). Interestingly, in validation of a large cohort, *LMBRD2* was significantly downregulated in SLE patients (*n* = 77) compared with healthy controls (*n* = 24) (Supplementary Figure 2A, *P* = 0.0017). Additionally, its expression is significantly positively correlated with *NONHSAT101022.2* (Supplementary Figure 2B, *r* = 0.5793, *P* < 0.0001). These results strongly suggest that *NONHSAT101022.2* may be involved in the pathogenesis of SLE by regulating *LMBRD2*. *LMBRD2* has a role in β2-adrenergic receptor (β2-AR) internalization, and knockdown of *LMBRD2* by siRNA can enhance β2-AR signal transduction sevenfold on stimulation by isoproterenol (Paek et al., 2017). The GO and KEGG enrichment analyses also suggested that *LMBRD2* is strongly related to the adrenergic receptor signaling pathway. NK cells express high levels of β2-AR (Scanzano and Cosentino, 2015), and stimulation with the β2-AR receptor reduces NK cell activity and cytotoxicity through the cAMP/PKA/p-CREB signaling pathway (Sun et al., 2018). In SLE patients, the number of NK cells is significantly reduced and its cytotoxicity is impaired (Park et al., 2009). However, Schepis et al. reported that the proportion of CD56^bright^ NK cells increased in the blood of active SLE patients (Schepis et al., 2009). The CD56^bright^ NK cells may form the main phenotype of PBMCs in active SLE patients, which reduces the cytotoxicity but increases the proportion of cells and the secretion of IFN-γ (Schepis et al., 2009; Hervier et al., 2011; Henriques et al., 2013). High IFN-γ levels are associated with the activity of SLE (Seery et al., 1997). Consistent with our results, higher NK cell levels were related to lower expression of *NONHSAT101022.2*, which was significantly related to the activity and severity of SLE. Therefore, we propose that the lncRNA *NONHSAT101022.2* may enhance the signal transduction of β2-AR by cis-regulating *LMBRD2*, which activates NK cells to produce high levels of IFN-γ, thereby exacerbating SLE. More experiments, both *in vitro* and *in vivo*, are needed to verify this hypothesis.

Next, we analyzed the sequencing results as a whole using a variety of bioinformatics methods. GO and KEGG enrichment analyses showed that the upregulated genes were mainly enriched in the immune and inflammation response and complement and coagulation cascades signaling pathway. These results are consistent with some of the known pathogenesis of SLE. SLE is caused by chronic and repeated activation of the immune system, accompanied by the production of antibodies and other protein products that contribute to inflammation and tissue damage (Han, 2012). Moreover, some defects of complement pathway gene products, including C2, C3, C4 and C1q, play an important role in the pathogenesis of lupus (Leffler et al., 2014; Macedo and Isaac, 2016). Interestingly, the TGF-β and JAK-STAT signaling pathways were significantly enriched for the downregulated genes. It has been reported that reduced serum and urine TGF-β1 levels are associated with renal damage in SLE patients (Jin et al., 2012; Vanarsa et al., 2020). Type I–II IFN, one of the pathogenic key signatures of SLE, triggers activation of the JAK-STAT pathway (Aue et al., 2020). This suggests that both pathways play an important role in the pathogenesis of lupus. Further experiments on the genes involved in these two pathways may shed more light on the pathogenesis of lupus.

Studies have found that the function of a lncRNA may be related to the genes with which it interacts (Quinodoz and Guttman, 2014; Engreitz et al., 2016; Yao et al., 2019). Another key role of lncRNAs is to act as miRNA sponges. Therefore, we constructed a lncRNA–miRNA–mRNA network based on the ceRNA hypothesis (Salmena et al., 2011). We have identified several verified miRNAs based on the ceRNA network, including *miR-150-5p*, *miR-128-3p* and *miR-146a-5p*, which are dysregulated in SLE patients and associated with the disease activity level of SLE (Su et al., 2018; van den Hoogen et al., 2018; Zeng et al., 2018). We propose that the *NONHSAT099004.2*–*miR-128-3p*–*ERG*, *MSTRG.85559.41*–*miR-150-5p*–*ITGB3* and *MSTRG.34071.2*–*miR-146a-5p*–*FAXDC2* axes may play stimulative roles in the disease activity and severity of SLE.

Gene Ontology and KEGG enrichment analyses of DETGs indicated that some of these lncRNAs are nuclear lncRNAs, which can act as key regulators of gene expression. All protein-coding genes and a large number of lncRNAs are transcribed by RNA polymerase II in eukaryotic genomes (Schier and Taatjes, 2020). In turn, lncRNAs can regulate RNA polymerase II activity by interacting with initiation complexes, thus exerting a transcriptional regulation function. lncRNAs can also regulate proteins by regulation of protein translation, transport and localization (Yao et al., 2019). *ADAM10*, one of the genes enriched in cellular protein localization, has been reported to mediate the cleavage of AXL receptor tyrosine kinase in PBMC of SLE patients, exacerbating the progression of lupus (Orme et al., 2016). Consequently, the lncRNA *MSTRG.23249.3* and its target gene *ADAM10* may be a regulatory axis involved in the progression of SLE. Moreover, we constructed a sub-network that consisted of 10 hub lncRNAs and 279 DETGs. These DETGs were mostly enriched in herpes simplex virus type 1 infection, the TGF-beta signaling pathway, and the intestinal immune network for IgA production. Viral infection, especially with Epstein–Barr virus, can lead to SLE through the type I interferon pathway (Han, 2012). Herpes simplex virus type 1 infection may also activate the type I interferon pathway similarly to Epstein–Barr virus, and lead to the occurrence of disease. The other signaling pathway, the intestinal immune network for IgA production, is enriched by IL-10, IL-15, and LTBR (lymphotoxin beta receptor). B cell abnormalities are another signature of SLE, which lead to the production of a large number of autoantibodies (such as IgG, IgM, and IgA) and cytokines (Dörner and Lipsky, 2016). IL-10 is a significant anti-inflammatory cytokine in autoimmune disease. It has been reported that B cells producing IL-10 can inhibit the development of lupus in a mouse model (Scapini et al., 2011). In our lncRNA expression profile, we found that *IL-10* is one of the target genes of the lncRNA *MSTRG.100048.9*. We suggest that the *MSTRG.100048.9*–*IL-10* axis is an important component of the abnormal activation of B cells in SLE.

In future studies, we will pay more attention to the *NONHSAT101022.2*–*LMBRD2*–β2-AR–NK Cell–IFN-γ axis, *NONHSAT099004.2*–*miR-128-3p*–*ERG*, *MSTRG.85559.41*–*miR-150-5p*–*ITGB3* and *MSTRG.34071.2*–*miR-146a-5p*–*FAXDC2* axes, *MSTRG. 23249.3*–*ADAM10*, and *MSTRG.100048.9*–*IL-10* axes, which will help us to better understand the mechanism of these lncRNAs involved in the pathogenesis of SLE. In addition, the verification and mechanism study of ten hub lncRNAs were also an important part of further study. We will also collect more PBMC samples from SLE patients and divide them into different cell subsets, such as T cells, B cells, NK cells and monocytes, for separate verification of the expression of these lncRNAs, so as to better understand their accurate cell localization and function. Another limitation of this paper is the relatively small number of samples used for validation. Large prospective cohort studies are also needed in future.

## Conclusion

In conclusion, we found many differentially expressed novel lncRNAs in PBMCs from patients with SLE. The function and regulatory mechanisms of these lncRNAs were analyzed by bioinformatic methods. The lncRNA *NONHSAT101022.2* is significantly downregulated in SLE patients and is also significantly related to the disease activity and severity. Additionally, we propose that *NONHSAT101022.2* may enhance the signal transduction of β2-AR by cis-regulating *LMBRD2*, which induces NK cells to produce high levels of IFN-γ, thereby exacerbating SLE.

## Data Availability Statement

The datasets presented in this study can be found in online repositories. The names of the repository/repositories and accession number(s) can be found below: GEO dataset and GSE162828.

## Ethics Statement

The studies involving human participants were reviewed and approved by Our study was approved by the Ethics Committee of The Second Affiliated Hospital of Zhejiang University School of Medicine (Approval Number: 2020-433). The patients/participants provided their written informed consent to participate in this study.

## Author Contributions

QC and YD did the conception and designed the study. QC, MC, XCn, XCa, YD, and HW performed the acquisition of clinical samples. QC carried out the data acquisition and analysis and drafted and wrote the manuscript. QC and MC performed the q-PCR verification experiment. YD and HW supervised and managed the data. YD reviewed and edited the manuscript and supported by funds. All authors have read and agreed to the published version of the manuscript and accept personal responsibility for the author’s contribution.

## Conflict of Interest

The authors declare that the research was conducted in the absence of any commercial or financial relationships that could be construed as a potential conflict of interest.

## Footnotes

1 https://david.ncifcrf.gov/home.jsp

2 http://www.webgestalt.org/

3 https://string-db.org

4 http://annolnc.gao-lab.org/index.php

## Supplementary Material

Supplementary Figure 1 Expression of two lncRNAs with no significance in 44 SLE patients and 24 healthy controls.

Supplementary Figure 2 Expression of *LMBRD2* and its correlation with *NONHSAT101022.2*.

## Abbreviations

SLE: systemic lupus erythematosus
DELs: differentially expressed lncRNAs
DEMs: differentially expressed mRNAs
PBMC: peripheral blood mononuclear cells
GO: Gene Ontology
KEGG: Kyoto Encyclopedia of Genes and Genomes
PCC: Pearson Correlation Coefficient
AUC: Area under the ROC curve
ROC: receiver operating characteristic curve
TGF-β: transforming growth factor-β
JAK-STAT: Janus kinase/signal transducer and activator of transcription
DETGs: differentially expressed target genes
SLEDAI: systemic lupus erythematosus disease activity index
ESR: erythrocyte sedimentation rate
CRP: C-reactive protein.
