# Supplementary material for: Novel Long Non-coding RNA Expression Profile of Peripheral Blood Mononuclear Cells Reveals Potential Biomarkers and Regulatory Mechanisms in Systemic Lupus Erythematosus
Source: Front Cell Dev Biol. 2021 Jun 2;9:639321. doi: 10.3389/fcell.2021.639321 (PMC8208038; doi:10.3389/fcell.2021.639321)
Supplement: Supplementary Figure 1 — Expression of two lncRNAs with no significance in 44 SLE patients and 24 healthy controls. [file Data_Sheet_1.docx]

Supplementary Material

**Supplementary Figure 1.** **Expression of two lncRNAs with no significance in 44 SLE patients and 24 healthy controls.** A. Expression of lncRNA ENST00000625135 has in two groups (*p=0.1822*). B. Expression of lncRNA MSTRG.44469.23 has no significance in two groups (*p=0.4286*).


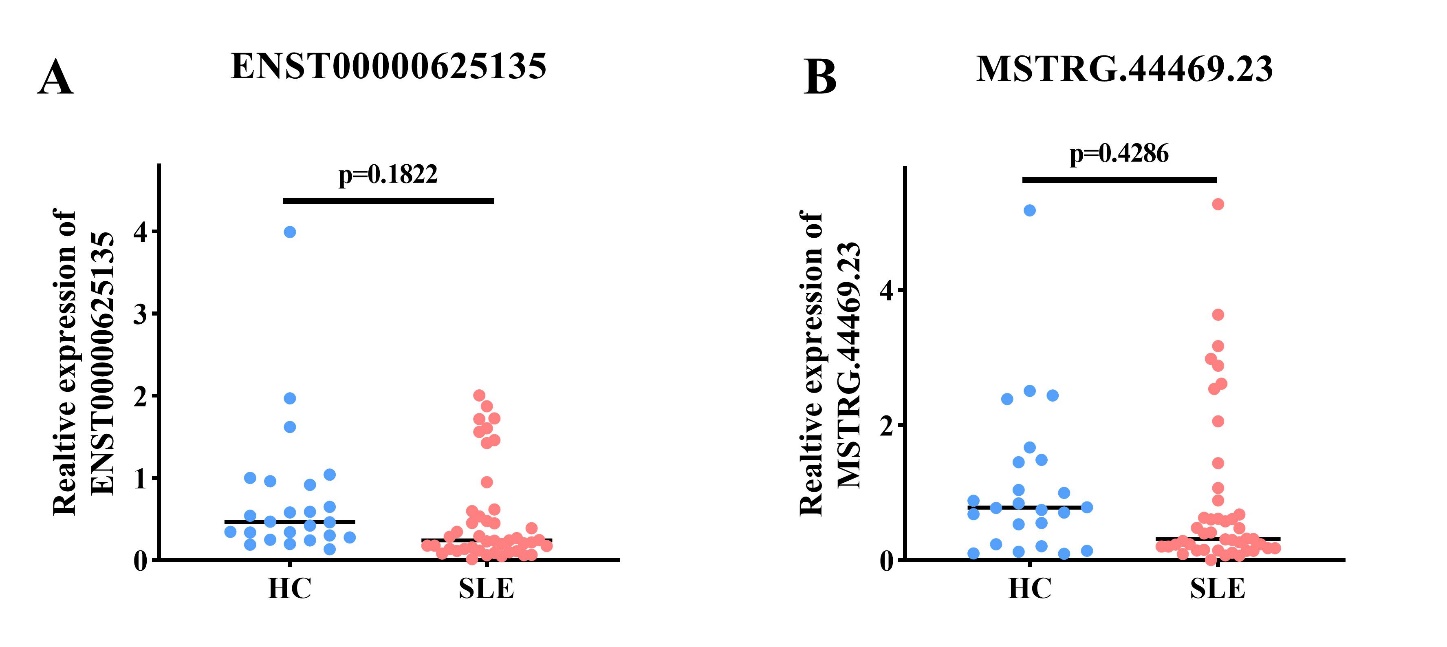


**Supplementary Figure 2. Expression of *LMBRD2 and* its correlation with *NONHSAT101022.2*.**

A. Expression of *LMBRD2* in 77 SLE patients and 24 healthy controls (*p*=0.0017). B. Correlation of *NONHSAT101022.2* and its target gene *LMBRD2* (*r* = 0.5793, *p* < 0.0001)

**
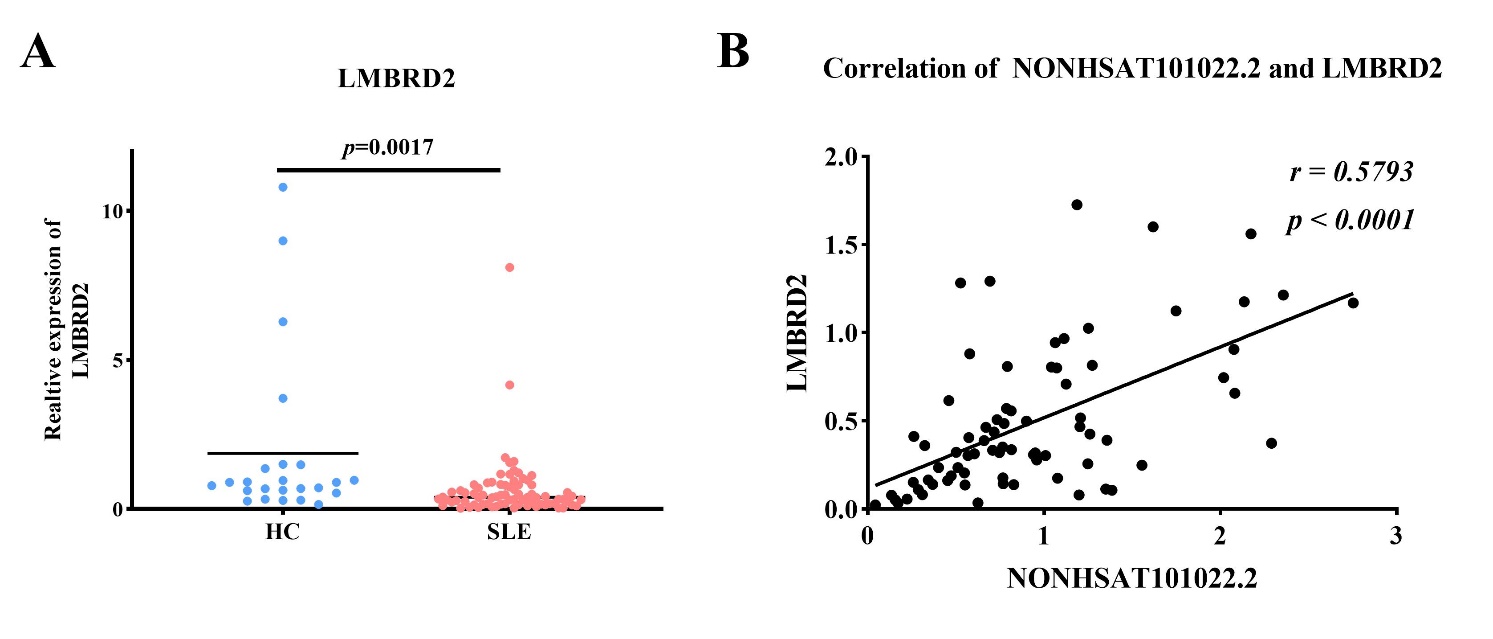
**
